# Exploration of the Protective Mechanism of Naringin in the Acetaminophen-Induced Hepatic Injury by Metabolomics

**DOI:** 10.1155/2022/7138194

**Published:** 2022-09-16

**Authors:** Zihan Lin, Guanzhen Wang, Wei Gu, Shengchao Zhao, Ziyi Shen, Wei Liu, Guodong Zheng, Baizhong Chen, Yi Cai, Mingxi Li, Chunpeng (Craig) Wan, Tingdong Yan

**Affiliations:** ^1^School of Life Sciences, Shanghai University, 99 Shangda Road, Shanghai 200444, China; ^2^University and College Key Lab of Natural Product Chemistry and Application in Xinjiang, School of Chemistry and Environmental Science, Yili Normal University, Yining 835000, China; ^3^Guangzhou Municipal and Guangdong Provincial Key Laboratory of Molecular Target & Clinical Pharmacology, the NMPA and State Key Laboratory of Respiratory Disease, School of Pharmaceutical Sciences and the Fifth Affiliated Hospital, Guangzhou Medical University, Guangzhou 511436, China; ^4^Guangdong Xinbaotang Biological Technology Co., Ltd., Guangdong, Jiangmen 529000, China; ^5^Research Center of Tea and Tea Culture, College of Agronomy, Jiangxi Agricultural University, Nanchang 330045, China

## Abstract

Naringin is a dihydroflavone which was found in citrus fruits. Previous studies have indicated the antiapoptotic, antioxidative stress, and anti-inflammatory effects of naringin. It can improve many common diseases, including fibrosis or hepatotoxicity, cardiovascular disease, and diabetes. Acetaminophen (APAP) is a frequently used painkiller, and hepatotoxic side effects limit its use. The purpose of the current examination is to find the impact of naringin on APAP-induced hepatic injury. Firstly, we pretreated mice model groups with naringin. Then, the liver injury model was established by injecting intraperitoneally into mice with APAP. After the mice were euthanized, we obtained serum and liver tissue samples from the mice. Finally, these samples were analyzed using a metabolomics approach to find the underlying mechanism of the effects of naringin on APAP-induced liver injury and provide a new treatment strategy for APAP-induced liver injury. Our data indicate that naringin significantly improves APAP-induced liver injury in mice and reduces the expression levels of liver injury markers in a dose-dependent manner. Furthermore, analysis of differential metabolites in mice with liver injury showed that naringin reduced APAP-induced hepatotoxicity due to reversing multiple metabolite expression levels and the rescue of energy, amino acid, and purine metabolism.

## 1. Introduction

Natural products are small molecular compounds extracted from fruits, vegetables, and Traditional Chinese medicine. Unlike traditional drugs, they generally have the characteristics of multiple efficacy and low toxicity and have potential therapeutic value for various complex diseases. Naringin, a naturally occurring and pharmacologically active flavonoid, was found in citrus fruits [1–5] such as lemons, grapefruits, sorbs, and vegetables [5]. Several studies have shown that this natural substance has a wide range of functions [2–4, 6], such as antioxidant, anti-inflammatory, and antiapoptotic roles. Further research has demonstrated that naringin can alleviate or improve a variety of common diseases, including fibrosis or hepatotoxicity [4, 6–9], cardiovascular diseases (CVD) [3, 10], diabetes [11], and neurodegenerative disease [12–14].

Some drugs have not been widely used in treating diseases because of severe side effects, including methotrexate (MTX) [4], and cyclophosphamide (CTX) [9]. Therefore, it is of great significance to find natural products that can alleviate the side effects of drugs. Elsawy et al. employed MTX to induce acute liver injury in rats and subsequently treated these rats with naringin. They found that naringin significantly reduced the upregulation of markers of MTX-induced liver injury and reduced oxidative stress, protecting hepatocytes from MTX-induced damage [4]. In another study on cyclophosphamide, naringin also alleviated CTX-induced oxidant production, hepatocyte dysfunction, and inflammation [9].

Acetaminophen (APAP) is frequently used as an analgesic agent whose hepatotoxic side effects are primarily involved in acute liver failure [15–17]. At safe doses, once ingested, APAP is quickly absorbed in the small intestine and immediately reaches inside liver cells. Enzymes catalyze the majority (about 80-90%) of APAP from the UDP-glucuronic acid transferase (UGT) 1A subfamily or sulfonate transferases. Then, it binds to glucuronic acid, forming nontoxic metabolites and excreting from the body in urine. Finally, this process does not damage liver cells [15, 16, 18, 19]. Whereas once the accumulation of APAP exceeds the tolerable dose for the human body, the glucuronidation and sulfonation pathways tend to saturate, and excessive APAP will be metabolized to form a large number N-acetyl-para-benzoquinoneimine (NAPQI). Unfortunately, NAPQI will deplete glutathione (GSH) stored by liver cells. Subsequently, redundant NAPQI react with biomacromolecules such as proteins, DNA, and unsaturated lipids in the cell, raising downstream events. For instance, reactive oxygen species (ROS) production, mitochondrial dysfunction, cell necrosis, apoptosis, autophagy, etc. cells will die in the end [15, 16, 18–20]. Hence, we can ameliorate the liver injury caused by APAP by targeting its metabolism.

Several previous studies have shown that naringin protects hepatocytes from liver injury by exerting antioxidant and anti-inflammatory effects [21–26]. Therefore, based on APAP metabolism, we are convinced that naringin may also be beneficial in improving APAP metabolism to reduce APAP-induced hepatotoxicity. Herein, a pretreated APAP-mediated liver injury mice model was exposed to naringin and then analyzed liver tissue and serum with metabolomic approaches to find whether naringin reduces APAP-induced hepatotoxicity and possible molecular mechanisms involved.

## 2. Materials and Methods

### 2.1. Ethical Statement

All procedures were performed by the current Chinese legislation on the care and use of laboratory animals and approved by the Department of Scientific Management of Guangzhou Medical University.

### 2.2. Naringin and Other Chemicals

All solvents mentioned in this article are LC-MS grade and purchased from Sigma-Aldrich. Ultrapure H_2_O was obtained from Milli-Q pure water system. The commercially available kits of alanine aminotransferase (ALT), aspartate aminotransferase (AST), glutathione peroxidase (GSH-Px), and malondialdehyde (MDA) are purchased from the Nanjing Jiancheng Bioengineering Institute (Jiancheng Bioengineering Institute, Nanjing, China). Naringin was bought from Shanghai Weihuan Biotechnology Co., Ltd. (Weihuan Biotechnology, Shanghai, China).

### 2.3. Centrifugal Operation

All centrifuge operations are performed by Dynamica centrifuges, purchased from the Shanghai Miaosheng Technology & Trade Co., Ltd. The model is Velocity 14R.

### 2.4. Animal Treatment

A total of 24 male C57BL/6J mice (age, 6 weeks) were obtained from Guangdong Medical Laboratory Animal Center (Guangzhou, China). Mice were randomly assigned into the following four groups: (1) vehicle control (*n* = 8), (2) APAP (*n* = 8), (3) APAP+50 mg/kg/d naringin (*n* = 8), and (4) APAP+100 mg/kg/d naringin (*n* = 8). Initially, naringin suspension was prepared from 0.5% carboxymethyl cellulose solution, and APAP was dissolved in normal saline at a concentration of 20 mg/ml and dissolved by heating at 55°C for animal experiment. The control group of these animal experiments was given the same volume of liquid but composed solely of the vehicle solution (0.5% carboxymethylcellulose). Naringin is a single treatment, and the mice were orally given naringin. Then, APAP (300 mg/kg) was injected intraperitoneally at 3 h following naringin treatment. About 48 h post-APAP exposure, mice were anaesthetized with pentobarbital (50 mg/kg, dissolved in saline with a concentration of 2%) via intraperitoneal injection. After that, the mice were sacrificed; tissue and serum samples were collected according to the instructions provided with commercial kits for subsequent experiments.

### 2.5. Serum ALT/AST Activity Analysis

The collected blood samples were placed at room temperature for about 2 h, centrifuge for 15 min at 840xg and separated serum stored at -20°C for later analysis. The serum alanine transaminase (ALT) and aspartate transaminase (AST) assays in each group were performed following the manufacturer's instructions.

### 2.6. Histopathology

The left liver lobe of the mice was fixed using paraformaldehyde (4%), dehydrated, and embedded in paraffin. The liver sections (5 *μ*m) were prepared and stained using hematoxylin and eosin (H&E), and then, microscopy was done. The cell necrotic boundaries were manually counted in three random fields per sample.

### 2.7. Glutathione Peroxidase and Malondialdehyde Measurement

The contents of glutathione peroxidase (GSH-Px) and malondialdehyde (MDA) in the liver tissues were assayed using commercial kits following the manual instructions provided by the manufacturers.

### 2.8. ^1^H NMR Analysis

The collected tissue samples were cut and weighed and consequently homogenized with 50% acetonitrile. The mixture was centrifuged at 12000xg for 10 min to remove the precipitate at 4°C. The supernatant was lyophilized under nitrogen, placed overnight in a -80°C refrigerator, and freeze-dried. The samples were placed in 550 *μ*l 99.8% D_2_O phosphate buffer (0.2 M, containing 0.05% sodium 3-(trimethylsilyl) propionate-2, 2, 3, 3-d4 (TSP), pH 7.4) and mixed with oscillation. The precipitation was removed by centrifugation. The supernatant was put on the machine for NMR analysis.

### 2.9. Data Preprocessing and Software

The TopSpin software (Bruker Biospin, Germany) version V3.0 was used to perform Fourier transform (FT), phase adjustment, baseline correction, and calibration. All spectra were multiplied by an exponential window function with a widening factor of 1 Hz when the Fourier transform was performed to improve SNR. The single-peak calibration of TSP at 0.00 ppm was used to remove the residual water peak, and the integral interval was 0.650-4.550 and 5.175-9.700 ppm. The integral interval was 0.015 ppm for data analysis.

The displacement range of finger-recognized compounds was imported into R software to calculate the integral area of each compound, and the fold value was obtained by intergroup comparison. Combined with a one-way analysis of variance, 0.05 was set as the threshold to screen differentially expressed metabolites.

### 2.10. Statistical Analysis

Data are presented as mean ± SEM. Statistical analysis was performed with the use of SPSS statistic software 15.0. Differences among groups were tested by one-way analysis of variance (ANOVA) with Tukey's post hoc test. In all cases, differences were considered statistically significant with *P* < 0.05.

## 3. Results

### 3.1. Naringin Pretreatment Repaired Liver Injury and Reversed the Level of Liver Injury Markers in the Samples

**Figure 1:**
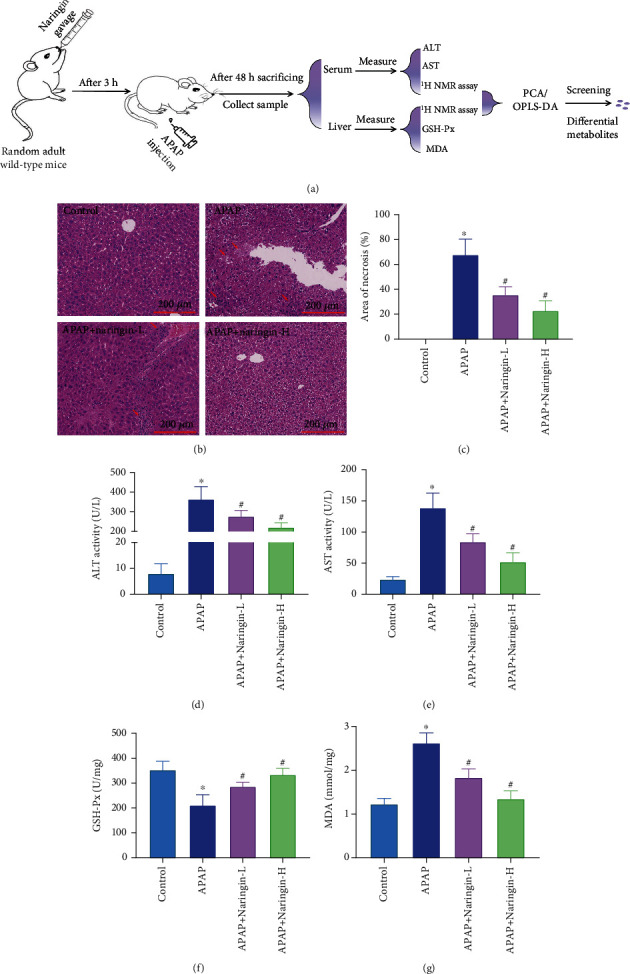
Naringin alleviates acetaminophen-induced hepatic injury in mice. (a) The design scheme of mouse experiment. (b) Hematoxylin-eosin (H&E) staining of mouse liver in control, APAP, APAP+naringin low-dose, and APAP+naringin high-dose groups. (c) Area of necrosis (%) in control, APAP, APAP+naringin low-dose, and APAP+naringin high-dose groups. (d-g) Histogram of changes of liver injury markers in control, APAP, APAP+naringin low-dose, and APAP+naringin high-dose groups. ^∗^*P* < 0.05 vs. control, ^#^*P* < 0.05 vs. APAP, *n* = 8.

We established a mouse model of liver injury using APAP and measured the extent of liver injury, including the proportion of liver cell necrosis and the production of liver injury markers. The proportion of liver injury and necrosis can be intuitively revealed by H&E staining results, whereas various markers can indirectly reflect APAP-induced endogenous metabolite changes. For this purpose, we selected four classic biomarkers for liver injuries, such as ALT, AST, MDA, and GSH-Px. The design scheme of the animal experiment is well elucidated (Figure 1(a)). Compared with the control, histopathology results of the liver tissue samples of mice in the model group showed that the liver tissue was significantly damaged after intraperitoneal injection of APAP (Figure 1(b)). In addition, statistics on the proportion of necrotic area showed that about 70% of the tissue was necrotic in the model group. After low-dose (50 mg/kg/d) naringin treatment, the necrotic area was reduced by about half, while high-dose (100 mg/kg/d) naringin treatment showed only about 20% necrotic area (Figure 1(c), *P* < 0.05). These results indicated that naringin repaired APAP-induced liver tissue damage and showed extremely significant therapeutic effects at high doses.

Furthermore, ALT and AST levels in serum and GSH-Px and MDA in liver tissue were measured. The results showed that naringin pretreatment reversed the levels of these markers of liver injury in the model group. To be specific, the levels of ALT, AST, and MDA in samples of model mice were significantly increased compared to the control group. Nevertheless, following pretreatment with naringin, ALT, AST, and MDA levels downregulated considerably in a dose-dependent fashion (Figures 1(d), 1(e), and 1(g), *P* < 0.05). Instead, although GSH-Px levels in the liver of model mice were significantly downregulated, naringin treatment reversed them (Figure 1(f), *P* < 0.05).

### 3.2. Identification of Major Metabolites in the Serum and Liver of Mice by NMR Spectroscopy

**Figure 2:**
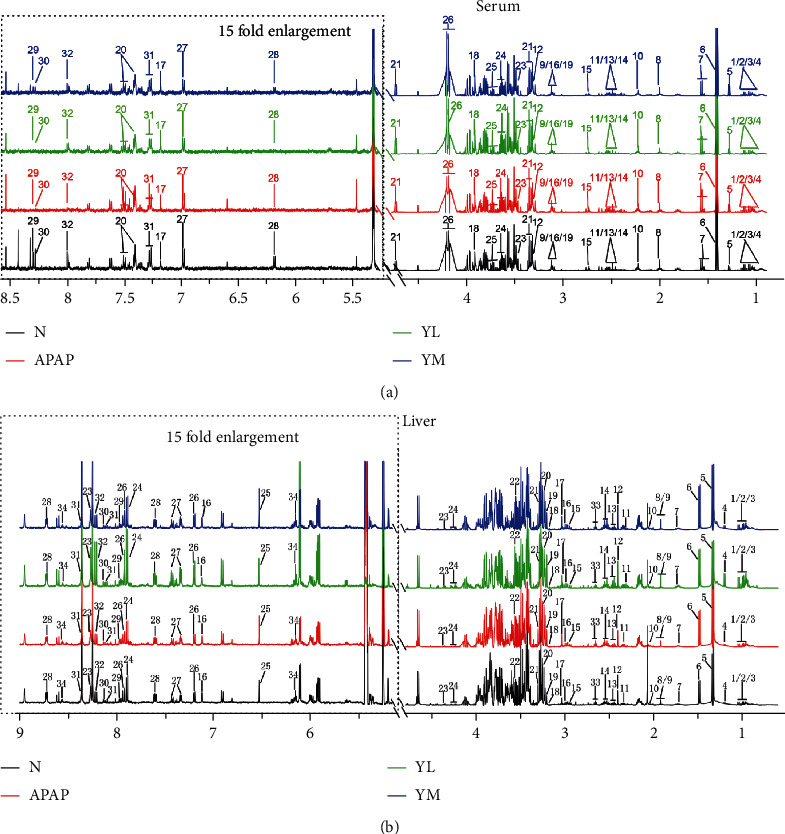
Typical 500 MHz CPMG ^1^H NMR spectra for serum (a) and liver (b) samples of the four groups of mice. N: control; APAP; YL: APAP+low-dose therapy; YM: APAP+high-dose therapy. Keys: 1. isoleucine; 2. leucine; 3. valine; 4. 3-hydroxyisobutyrate; 5. 3-hydroxybutyrate; 6. lactate; 7. alanine; 8. acetate; 9. lysine; 10. homoserine; 11. glutamine; 12. O-acetylcarnitine; 13. pyruvate; 14. succinate; 15. citrate; 16. N, N-dimethylglycine; 17. histamine; 18. creatine; 19. ornithine; 20. phenylalanine; 21. glucose; 22. taurine; 23. methanol; 24. glycerol; 25. threonine; 26. 1,3-dihydroxyacetone; 27. tyrosine; 28. inosine; 29. formate; 30. hypoxanthine; 31. tryptophan; 32. xanthine.

After ^1^H, NMR experiments were conducted on the serum and liver samples of the four groups of mice (control group, marked as N; APAP group, marked as APAP; APAP+low-dose naringin therapy group, marked as YL; APAP+high-dose naringin therapy group, marked as YM), the typical hydrogen spectra as shown in Figure 2 were obtained. The names are identified by querying public metabolomics databases, such as HMDB (http://www.hmdb.ca/) and MMCD (http://mmcd.nmrfam.wisc.edu/), and annotated in legends.

29 and 28 metabolites were identified in extracts of serum (Figure 2(a)) and liver (Figure 2(b)), respectively. To visualize the identification results, different colors and numbers represent different groups of spectra and identified metabolites, respectively. In addition, based on the low metabolite concentrations in the low-field region (2.175-9.700 ppm), the spectra were magnified by a factor of 15 to align with the high-field region.

### 3.3. Naringin Can Reverse the Level of Small Molecule Metabolites in Serum and Liver Tissue Induced by APAP in Mice

#### 3.3.1. PCA

**Figure 3:**
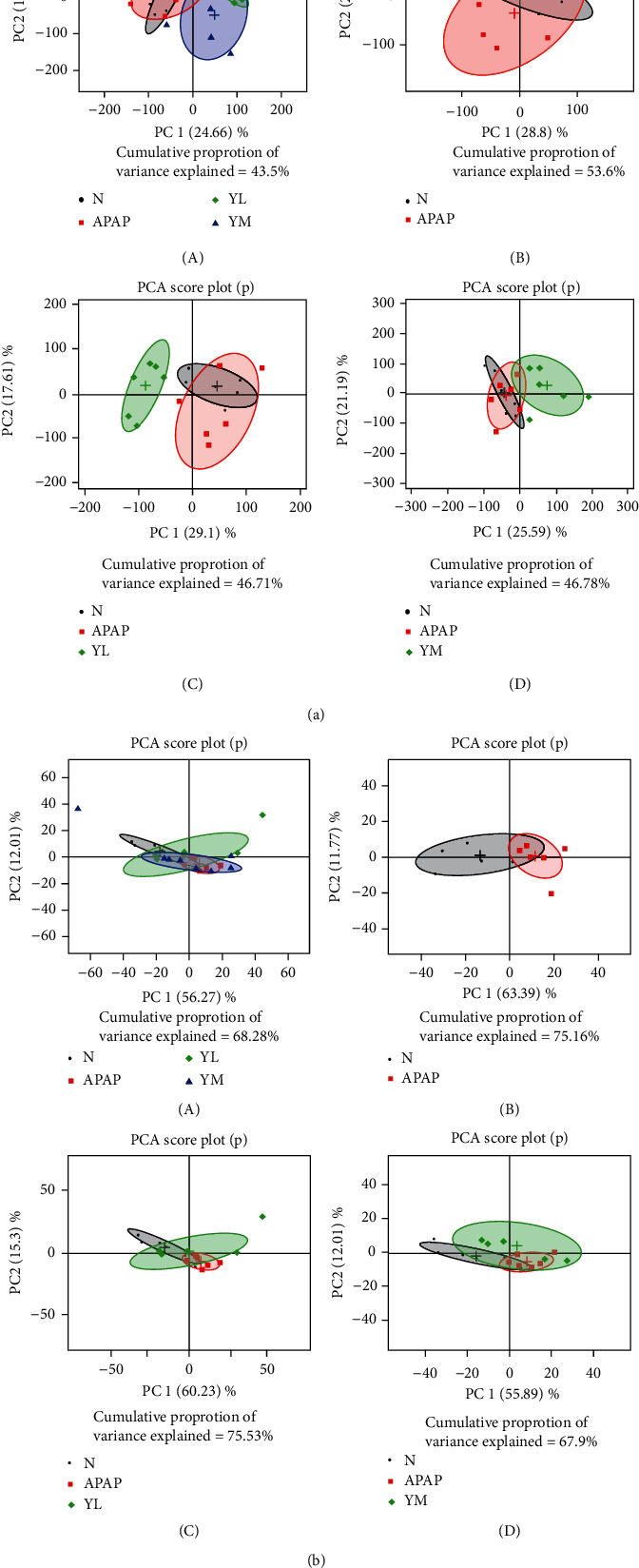
PCA score plots. (a) PCA score plot based on ^1^H CPMG NMR spectra of serum obtained from four groups. (b) PCA score plot based on ^1^H CPMG NMR spectra of liver obtained from four groups. N: control; APAP; YL: APAP+low-dose therapy; YM: APAP+high-dose therapy.

We used R software to carry out pattern recognition multivariable analysis for the normalized spectral data. We used the mean centre scaling method to obtain the PCA (principal component analysis) graph, shown in Figure 3, to describe the weight of principal components. These graphics can help us analyze the changes of small molecule metabolites in serum and liver of APAP-induced mice compared to the control group. PCA patterns based on the NMR spectra of mouse serum are shown in Figure 3(a). The APAP-administered group and the control group mostly overlapped, indicating that APAP did not change the overall metabolic level of the mouse serum (Figure 3(a)-A and B). However, the drug-treated groups (YL/YM) showed a separation trend with the control group, suggesting that naringin changed metabolites levels in serum(Figures 3(a)-A, C, and D, the green figure has no overlap with the grey figure). In the liver, both APAP and drug-treated groups overlapped completely with the control group, indicating that neither APAP nor drugs caused liver metabolite disturbances (Figure 3(b)). However, PCA is an unsupervised analytical method, and to further differentiate metabolic levels between groups, we used the supervised OPLS-DA to filter out irrelevant factors.

#### 3.3.2. OPLS-DA

**Figure 4:**
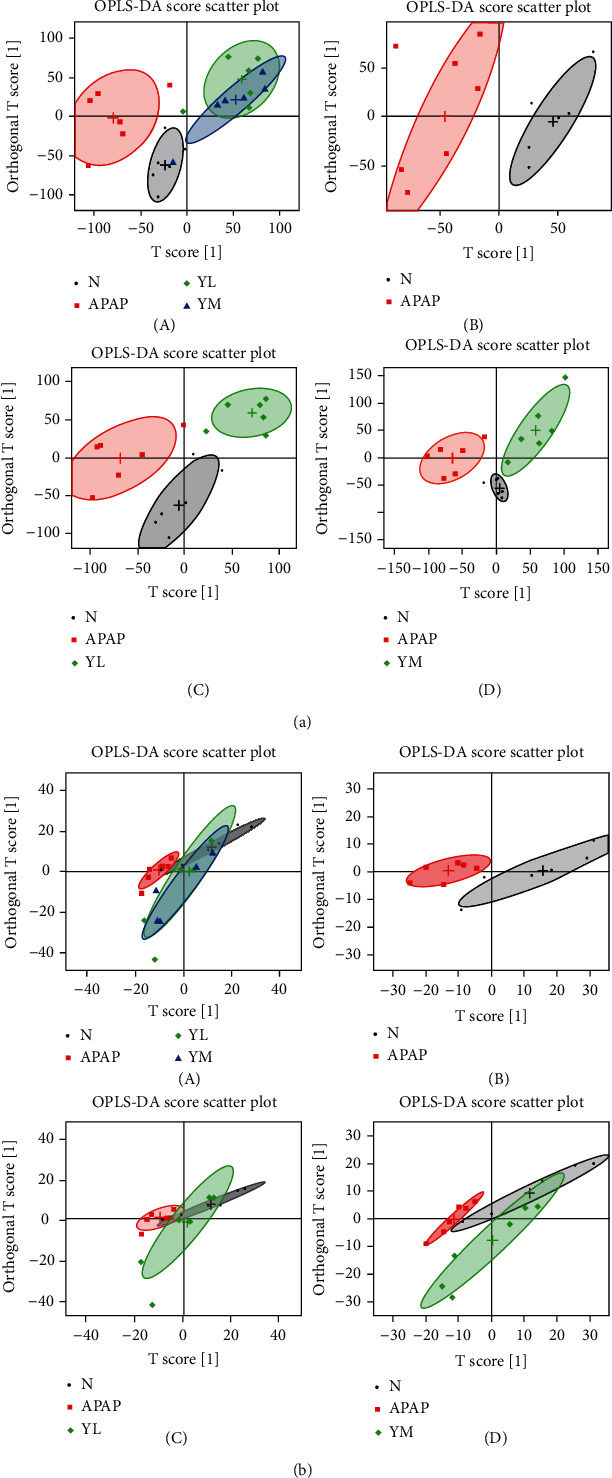
OPLS-DA score plots. (a) OPLS-DA score plots (a, b, c, and d), respectively, are derived from ^1^H NMR spectra of serum from different groups. (b) OPLS-DA score plots (a, b, c, and d), respectively, are derived from ^1^H NMR spectra of liver from different groups. N: control; APAP; YL: APAP+low-dose therapy; YM: APAP+high-dose therapy.

The OPLS-DA score chart is shown in Figure 4. Based on the complete separation of APAP and control groups, metabolic disturbances in serum were caused by APAP (Figure 4(a)-B). However, compared with the APAP group, the administration group was closer to the control group, especially the YM group, indicating that the metabolic disturbance caused by APAP was reversed in a dose-dependent manner (Figure 4(a)-A). Consistent with the serum results, the APAP group was completely separated from the control group (Figure 4(b)-B). In contrast, the drug and control groups overlapped completely (Figure 4(a)-A, C, D). The results indicated that APAP caused the disturbance of the overall metabolic level of the liver, but was improved by the drug.

### 3.4. Screening Small Molecule Metabolites with Significant Differences

**Figure 5:**
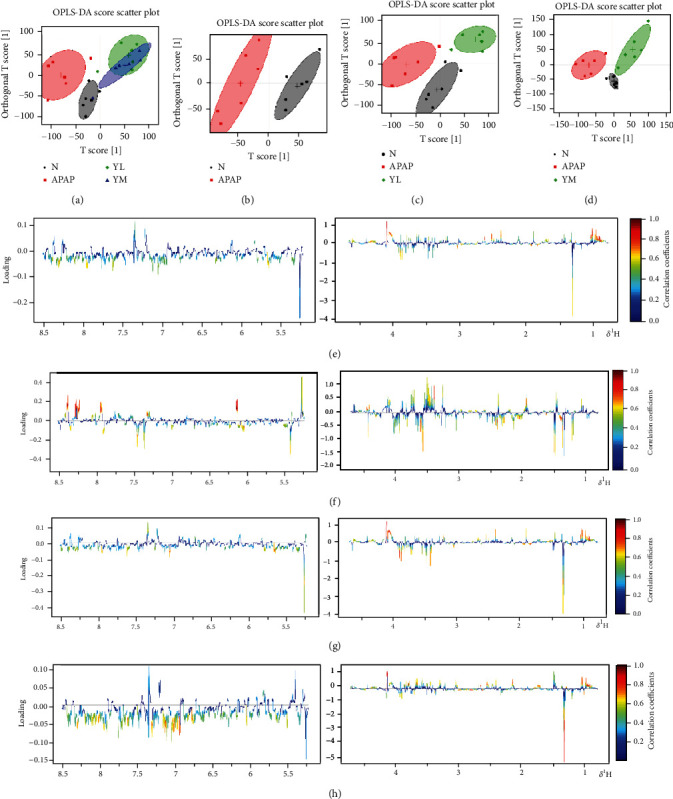
OSC-PLS-DA score plots (a–d) derived from the ^1^H NMR spectra of serum and corresponding coefficient color-coded loading plots (e–h) obtained from different groups. The color map shows the significance of metabolite variations between the two classes. The color bar corresponds to the weight of the corresponding variable in the discrimination of statistically significant (red) or no significant (blue). Positive and negative peaks indicate a relatively decreased and increased metabolite level in different groups. Peaks in the positive direction indicate metabolites that are more abundant in the groups in the positive direction of the first principal component. Consequently, metabolites that are more abundant in the groups in the negative direction of the first primary component are presented as peaks in the negative direction. Keys of the assignments were shown in Figure 2(a). N: control; APAP; YL: APAP+low-dose therapy; YM: APAP +high-dose therapy.

**Figure 6:**
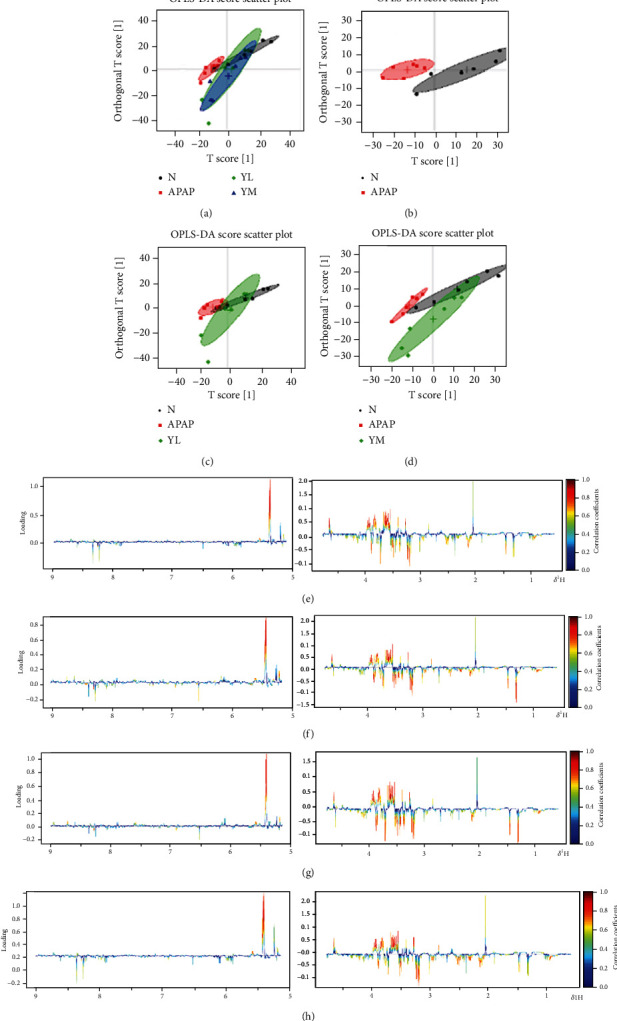
OSC-PLS-DA score plots (a–d) derived from the ^1^H NMR spectra of liver and corresponding coefficient color-coded loading plots (e–h) obtained from different groups. The color map shows the significance of metabolite variations between the two classes. The color bar corresponds to the weight of the corresponding variable in the discrimination of statistically significant (red) or no significant (blue). Positive and negative peaks indicate a relatively decreased and increased metabolite level in different groups. Peaks in the positive direction indicate more abundant metabolites in the groups in the positive direction of the first principal component. Consequently, metabolites that are more abundant in the groups in the negative direction of the first primary component are presented as peaks in the negative direction. Keys to the assignments are shown in Figure 2(b). N:control; APAP; YL: APAP+low-dose therapy; YM: APAP+high-dose therapy.

To further identify the differential metabolites, the loading value of the first principal component of the OSC-OPLS-DA model was adopted to plot the loading plot, as shown in Figures 5 and 6. Orthogonal signal correction (OSC) was performed on the PLS-DA model. OSC could remove the variables that were not meaningful to the grouping to maximize the differences between groups. To find the differential metabolites contributing to group separation, the redder the peak, the more significant difference between the two groups, and the bluer the peak, there is no significant difference. The peak above the loading plot has a higher content in the group on the right side of the OPLS-DA score, while the peak below the loading plot has a higher content on the left side OPLS-DA score. The displacement range of finger-recognized compounds was imported into R software to calculate the integral area of each compound, and the fold value was obtained by intergroup comparison. Combined with a one-way analysis of variance, 0.05 was set as the threshold to screen differentially expressed metabolites. Please refer to Tables 1 and 2 for details.

According to the information on differential metabolites, the reduced metabolites (isoleucine, valine, 3-hydroxyisobutyrate, acetate, pyruvate, histamine, inosine, hypoxanthine, and xanthine) can be observed in model groups compared to control groups in serum samples. Metabolites with elevated expression levels include leucine, 3-hydroxybutyrate, alanine, homoserine, glutamine, taurine, glycerol, threonine and 1, 3-dihydroxyacetone. The significant decrease in pyruvate, inosine, hypoxanthine, and xanthine, while 3-hydroxybutyrate, alanine, homoserine, taurine, and threonine is significantly increased. However, although APAP treatment caused this change in metabolites, there is a certain degree of reversal effect after naringin treatment and presented the characteristics of dose-dependence.

Similarly, there were reduced metabolites in the APAP model group in liver tissue samples compared to the control group, including 3-hydroxyisobutyrate, ethanolamine, and ATP. The significantly increased metabolites are leucine, lactate, alanine, glutamate, isocitrate, creatine, choline, and fumarate. Naringin treatment also reversed the expression levels of some metabolites, including ATP, leucine, alanine, and fumarate, in a dose-dependent manner. However, most metabolite changes did not improve with medium doses of naringin, suggesting that further research is needed to determine the therapeutic dose of naringin to achieve the best protective effect.

### 3.5. Summary of Pathway Analysis

**Figure 7:**
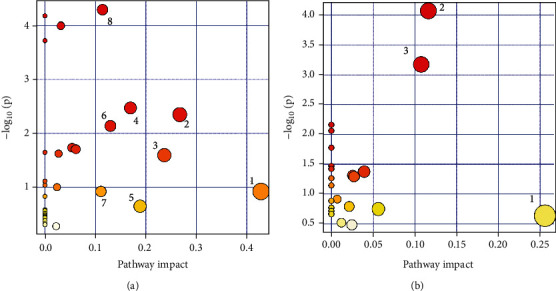
Summary of pathway analysis of serum and liver. (a) Serum: (1) taurine and hypotaurine metabolism; (2) pyruvate metabolism; (3) glycerolipid metabolism; (4) histidine metabolism; (5) citrate cycle (TCA cycle); (6) glycolysis/gluconeogenesis; (7) alanine, aspartate, and glutamate metabolism; (8) arginine and proline metabolism. (b) Liver: (1) glutathione metabolism; (2) alanine, aspartate, and glutamate metabolism; (3) citrate cycle (TCA cycle).

In addition, to reveal metabolic pathways that may be involved in naringin improving liver injury, metabolic pathway analysis of differential metabolites of serum and liver between normal and liver-injured mice was performed using MetaboAnalyst 5.0. The results are shown in Figure 7. In general, pathways with an impact value of >0.1 was considered potentially targeted metabolic pathways. As shown in Figure 7, naringin treatment significantly affected the metabolism of various amino acids, TCA cycle, and gluconeogenesis pathways in serum and liver. These data suggest that naringin may play its role in protecting liver cells by targeting these metabolic pathways.

## 4. Discussion

Naringin has antioxidant, antiapoptotic, and anti-inflammatory effects in several studies. It is found that naringin protects endothelial cells from ox-LDL-induced apoptosis and injury in an in vitro model of atherosclerosis [3].

In this study, we were interested in whether naringin could affect APAP-mediated liver injury, providing new ideas for the treatment or remission of this hepatotoxicity. APAP is an essential factor in the cause of acute liver failure, and this nature has seriously affected the use of this drug. Therefore, people should explore a new substance to alleviate this side effect. There have been some studies on APAP metabolism, but there is no way to avoid this side effect altogether. The challenge is that APAP metabolism involves a wide range of pathways. To this end, we investigated the impact of naringin on APAP-induced hepatotoxicity and the possible molecular mechanisms engaged by analyzing the degree of metabolite changes induced by APAP.

The liver is the chief organ controlling and managing the metabolism in the body, equipped with a variety of metabolic enzymes, including AST and ALT, primarily involved in catalyzing the transfer of amino acids between amino acids and ketone acids [27]. However, liver cells damage caused by external stimulation may disturb the integrity of the cellular membranes and enhance the permeability. This increased permeability enhances ALT and AST release into the blood to raise blood serum levels of ALT and AST. Therefore, the detection of blood ALT and AST levels indirectly reflects the degree of liver injury. The liver serves as a major site for biological oxidation; when aerobic cells are metabolized, a burst of reactive oxygen species (ROS) is generated that results in lipid peroxidation of cell membranes and damage to surrounding cells in the liver tissues. ROS-mediated lipid peroxidation elevated MDA levels, and fluctuating levels of MDA may reflect the degree of lipid peroxidation in liver cells [28].

On the contrary, the primary function of GSH-Px is to decompose peroxides. Therefore, when liver cells are damaged, the activity of GSH-Px is reduced, and the peroxides cannot be decomposed, and the antioxidant capacity of the liver is reduced [29]. Our results have demonstrated that, compared with normal mouse samples, model mice have significantly increased areas of liver necrosis and significantly increased levels of ALT and AST in serum and MDA in the liver, which naringin treatment successfully reversed in a dose-dependent manner. As expected, APAP-induced liver injury decreased GSH-Px activity, whereas high-dose naringin significantly enhanced GSH-Px activity. On the other hand, R software was used to perform orthogonal partial least squares (OPLS) on normalized data to find the correlation between NMR data (the *x* variable) and other variables (*Y* variable, grouping information). Partial least squares discriminant analysis (PLS-DA) uses the data scale scaling method of unit variance scaling. The PLS-DA was used to test the quality of the model with the two-fold cross-validation method, and the R2X and Q2 obtained after the cross-validation were used to evaluate the validity of the model (representing the variables that could be explained by the model and the predictability of the model, respectively). After that, the model's effectiveness was further tested by changing the order of the classification variable *Y* randomly several times (*n* = 2000) to obtain the corresponding random *Q*2 value. OPLS-DA filtered out irrelevant orthogonal signals to obtain more reliable differential metabolites. PCA graph and OPLS-DA score plots were used to analyze the experimental results of ^1^H NMR, which showed that induction of APAP did change the level of small molecule metabolites in the samples, and the expression was more evident in liver samples. However, treatment with naringin saved the levels of metabolites in the model samples, suggesting that naringin has the potential to treat APAP-induced liver injury.

The levels of succinate in serum and fumarate in the liver of the APAP model group were significantly increased, while ATP (Adenosine triphosphate) level was significantly decreased. Both succinate and fumarate are important intermediates of TCA circulation and the respiratory chain. Thus, their increase indicates that the APAP group mice have damaged mitochondria, disrupted TCA circulation, and disrupted the respiratory chain, and reduced efficiency of energy generation [30]. ATP is the main molecule that drives the cellular reaction process. Glucose is converted to pyruvate, and two ATP molecules are produced through glycolysis. Subsequently, when oxygen is sufficient, pyruvate is directed into the tricarboxylic acid cycle, where a reaction involving substrate-level phosphorylation can have a certain amount of ATP. The respiration chains can also phosphorylate the intermediate to produce large amounts of ATP [31]. In contrast, when oxygen is scarce, pyruvate is converted to lactate, a process that produces little ATP. Consistent with this, there was a significant increase in lactate in serum and liver in the APAP group, indicating an enhanced anaerobic glycolysis pathway that ultimately leads to inadequate ATP supply. Therefore, to cope with this energy crisis, the body needs to use other ways to produce energy. Fatty acid oxidation can release a large amount of energy. As a product of fatty acid oxidation [32], acetate was significantly upregulated in the liver of the APAP model group, indicating that the fatty acid oxidation process was enhanced.

Gluconeogenesis is another energy-saving pathway in which enzymes gradually convert pyruvate into glucose, providing the body with more glucose for energy production. A precursor of gluconeogenesis [33], alanine, is a key link between carbohydrate and amino acid metabolism. In this cycle, the ammonia produced by the breakdown of proteins is converted to L-alanine by transamination, which is then transported to the liver. Catalyzed by alanine aminotransferase, the amino group is transferred to *α*-ketoglutarate to form glutamate and pyruvate, which can be further converted to glucose [34]. However, our data showed that alanine levels in serum and liver of the APAP model group were significantly increased, while pyruvate and glucose levels were not significantly increased, indicating that the gluconeogenesis pathway was impaired. Therefore, fatty acid oxidation may be the leading energy supplement for APAP-induced stimulation in mice in the model group.

Furthermore, liver samples from the APAP model group showed significantly elevated leucine, isoleucine, and valine levels. They are commonly known as branched-chain amino acids essential for protein synthesis [35–37]. As previously mentioned, induction of APAP produces ROS, which further destroys proteins in the cytoplasm and membranes of liver cells [38]. The elevated levels of these three essential amino acids indicate enhanced hepatic catabolism and damage to some proteins. Our results suggest that low-dose naringin therapy salvages the increased levels of branched amino acids preventing ROS damage to proteins.

A purine catabolic pathway can be performed to save the body from oxidative damage. In this process, inosine goes through the first step of metabolism to produce hypoxanthine, which is then converted to xanthine by the catalysis of xanthine oxidoreductase and finally converted from xanthine to uric acid, which is an effective free radical scavenger with antioxidant effect [30, 39]. Therefore, the significant reduction of inosine, hypoxanthine, and xanthne in the APAP model group may be attributed to a self-protection mechanism of hepatocytes.

In conclusion, our study demonstrates the potential value of naringin in the treatment of APAP-induced liver injury in mice. The underlying mechanisms include antioxidants, salvage, glucose metabolism, regulation of amino acids, and purine metabolism. Our study focused on the changes of endogenous metabolites in mice with liver injury and explored the therapeutic effects of naringin based on them. Using metabolomics analysis based on NMR, we identified small molecule metabolites that changed significantly before and after drug treatment and explored the metabolic pathways involved, providing a new strategy for alleviating the liver toxicity and side effects of APAP drugs.

## Figures and Tables

**Table 1:** Differential expression of metabolites between each two groups in serum.

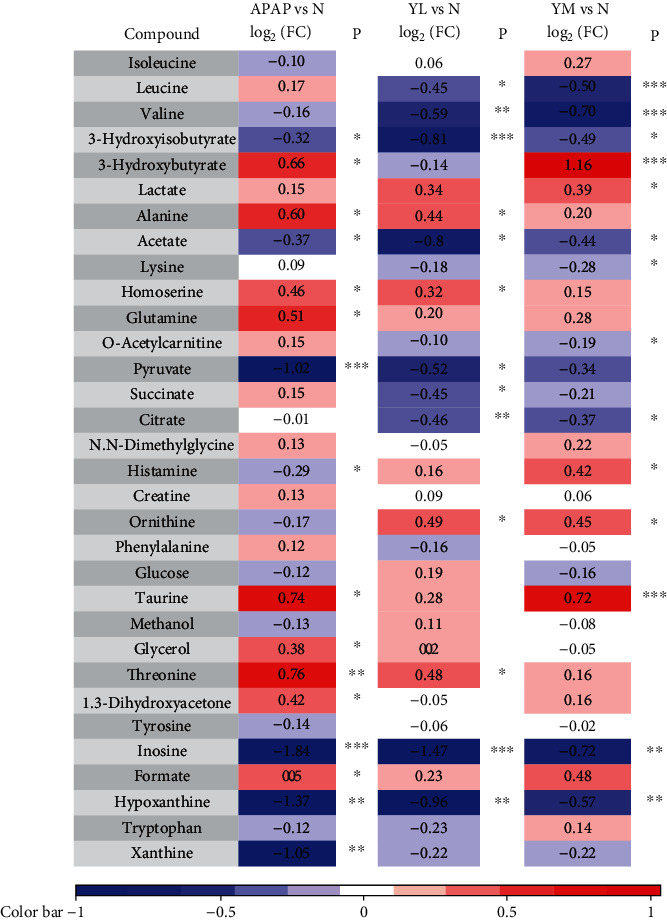

^a^Color coded according to the logarithmic transformation of fold change (log_2_(FC)), warm color (red) shows the degree of increase in identified metabolites, and blue the decrease in APAP vs. N, YL vs. N, and YM vs. N groups. ^b^*P* values corrected by Benjamini Hochberg methods were calculated based on a parametric Student's *t*-test or a nonparametric Mann–Whitney test. ^∗^*P* < 0.05, ^∗∗^*P* < 0.01, ^∗∗∗^*P* < 0.001.

**Table 2:** Differential expression of metabolites between each two groups in liver.

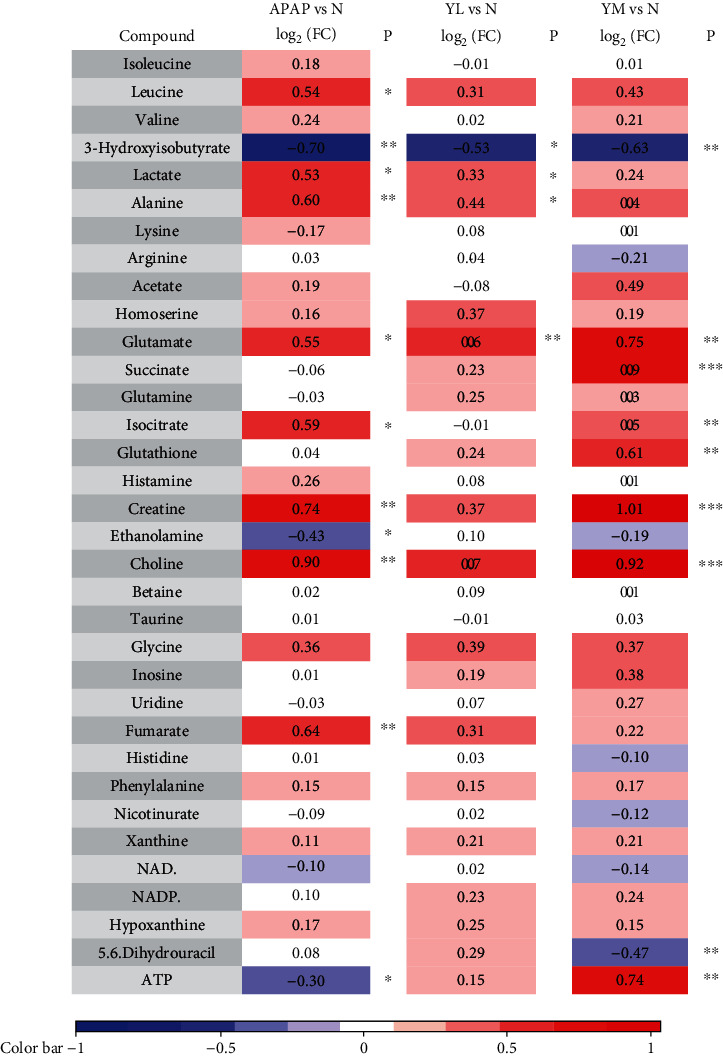

^a^Color coded according to the logarithmic transformation of fold change (log_2_(FC)), warm color (red) shows the degree of increase in identified metabolites, and blue the decrease in APAP vs. N, YL vs. N, and YM vs. N groups. ^b^*P* values corrected by Benjamini Hochberg methods were calculated based on a parametric Student's *t*-test or a nonparametric Mann–Whitney test. ^∗^*P* < 0.05, ^∗∗^*P* < 0.01, ^∗∗∗^*P* < 0.001.

## Data Availability

The original contributions presented in the study are included in the article. Further inquiries can be directed to the corresponding authors.
